# The Motif of ^76^KRKCSK in Bm65 Is an Efficient Nuclear Localization Signal Involved in Production of Infectious Virions

**DOI:** 10.3389/fmicb.2019.02739

**Published:** 2019-11-26

**Authors:** Guohui Li, Xinyu Qi, Huiqing Chen, Zhaoyang Hu, Fangying Chen, Liang Deng, Zhongjian Guo, Keping Chen, Qi Tang

**Affiliations:** Institute of Life Sciences, Jiangsu University, Zhenjiang, China

**Keywords:** *Bm*NPV Bm65, Bm65 tetramer, point mutation, Bm65 truncation, nuclear localization signal

## Abstract

*orf65 (Bm65)* of Bombyx mori nucleopolyhedrovirus (BmNPV) codes for a putative 104-amino-acid protein containing three cysteine residues with a putative molecular mass of 12.2 kDa. Previous studies have showed that Bm65 accumulates mainly in nucleus and involved in the repair of UV-damaged DNA. However, the mechanism of nuclear import of Bm65 remains unclear. In this study, a SDS-stable Bm65 tetramer was found in BmNPV-infected BmN cells, and alanine substitutions for the three cysteine residues did not affect the formation of Bm65 tetramer. Additionally, a basic amino acid cluster of the Bm65 protein was identified as an efficient nuclear localization signal (NLS). Firstly, transient expression of GFP-fused truncated Bm65 variants revealed that the ^76^KRKCSK motif functions as the NLS. This was also confirmed by alanine substitution in the ^76^KRKCSK motif, which caused attenuated nuclear localization of Bm65. Next, the ^76^KRKCSK motif-mutated bacmid was generated and the ^76^KRKCSK motif was also found to be important for nuclear localization of Bm65 in BmNPV-infected conditions. Lastly, analyses of flag-tagged Bm65 expressing bacmids revealed that the mutations in ^76^KRKCSK motif did not affect the synthesis of Bm65 tetramer, but severely impaired production levels of infectious virions. In conclusion, Bm65 exists in mainly a tetrameric form in virus-infected cells, which may be involved with production levels of infectious virions.

## Introduction

Baculoviruses are within a group of enveloped, double-stranded DNA insect viruses with large, closed and circular genomes ranging in size from 80 to 180 kbp (Herniou et al., 2003; van Oers and Vlak, 2007; Yin et al., 2015), which are characterized by a biphasic infection phase with production of two types of progeny virions during the viral life cycle. The two phenotypes are budded viruses (BVs) and occlusion-derived viruses (ODVs), which carry identical genetic information but differ structurally and functionally (Slack and Arif, 2007; Cheng and Lynn, 2009). BVs are produced at the initial stage of baculovirus life cycle and are responsible for the spread of infection from cell to cell, while ODVs are produced in the late stage of viral life cycle and mediate the horizontal transmission among insects (Clem and Passarelli, 2013; Rohrmann, 2013).

Bombyx mori nucleopolyhedrovirus (BmNPV) belongs to the *Alphabaculovirus* genus, *Baculoviridae* family and infects exclusively silkworms. BmNPV epizootics result in serious losses in silk production. Therefore, it is necessary to clarify the mechanism of BmNPV infection at the molecular level, which is helpful to control viral spread among silkworms. The functions of most viral genes in the process of BmNPV propagation, interactions between BmNPV and silkworm, and the innate response against BmNPV invasion have been extensively studied since Gomi et al. (1999) published the sequence of BmNPV genome (Ono et al., 2012; Qin et al., 2012; Xue et al., 2012). Additionally, the mechanism of BmNPV proliferation in silkworm has been gradually elucidated. Like other viruses, the propagation of BmNPV in host cells is inevitably involved with an important number of virus-encoded proteins that are required to generate progeny virions. Previous research reported that Bm65 is an early gene by transcriptional analysis (Tang et al., 2013), indicating that Bm65 may be involved with viral propagation. Tang et al. (2015, 2017) further reported that Bm65 localizes mainly in nucleus and is involved with the repair of UV-damaged DNA. However, the size of Bm65 in BmNPV-infected conditions remains unclear. So, we want to check the expression of Bm65 in BmNPV-infected BmN cells. Meanwhile, the mechanism of nuclear import and the impact of Bm65 on viral propagation are demonstrated in the study.

In the current study, a series of transient expression plasmids, including Bm65 truncations and point mutations in Bm65, were fused with enhanced green fluorescent protein (EGFP) respectively. The target DNA fragments were under control of *ie1* promoter for expression of fusion protein tagged with EGFP. These plasmids were transfected into BmN cells to examine the intracellular distribution of fluorescent signal. Furthermore, the effect of mutations in Bm65 ^76^KRKCSK motif on viral propagation was further evaluated by analysis of production of infectious virions.

## Materials and Methods

### Bacmid, Virus, Plasmids, Bacterial Strains, and Cells

Bombyx mori nucleopolyhedrovirus (BmNPV) bacmid (Bm-bacmid) with a deletion of *Bm65* (Bm^Bm65KO^) was generated as previously described (Tang et al., 2013), and propagated in *Escherichia coli* strain DH10B harboring the pMON7124 helper plasmid. vBm^(PBm65–Bm65–egfp)^ was made by Tang et al. (2015) and used as a control of wild type in the study. Plasmid of pFastHTB-P_Bm__65_-Bm65-*egfp* was constructed as previously described by Tang et al. (2015). Recombinant plasmid pFastHTB-P_ie__1_-*ns1*-*egfp* constructed by Li et al. (2015) was used to construct serial *Bm65* truncations fused with *egfp* to study intracellular distribution of fluorescence signals in BmN cells. *E. coli* strains DH5α and DH10B were maintained in our laboratory. BmN cells were cultured at 27°C in TC-100 medium supplemented with 10% Gibco fetal calf serum (Life Technologies).

### Transient Expression Plasmids Used for Subcellular Localization of Bm65

Primer pair Bm65-F1 and Bm65-R was used to amplify the full length of *Bm65*, in which the TAA stop codon was deleted, and target DNA was subcloned into pFastHTB-P_ie__1_-*ns1*-*egfp* to generate pFastHTB-P_ie__1_-Bm65-*egfp*. Additionally, a series of 3′-terminally truncated Bm65 fragments were amplified from Bm-bacmid using different primer pairs as follows. Primer pair Bm65-F1 and Bm65-R1 were used to amplify *Bm65(T1)* with a 3′-terminal deletion of 60 bp; primer pair Bm65-F1 and Bm65-R2 were designed to amplify *Bm65(T2)* with a 3′-terminal deletion of 87 bp. Primer pair Bm65-F2 and Bm65-R were designed to amplify *Bm65(T3)* with a 5′-terminal deletion of 108 bp. Primer pair Bm65-F3 and Bm65-R were designed to amplify *Bm65(T4)* with a 5′-terminal deletion of 210 bp. Primer pair Bm65-F1 and Bm65-R3 were designed to amplify *Bm65(T5)* with a 3′-terminal deletion of 216 bp. The PCR products were respectively ligated into *Eco*RI- and *Pst*I- digested pFastHTB-P_ie__1_-*ns1*-*egfp* to generate the final plasmids. The final plasmids were named pFastHTB-P_ie__1_-Bm65 (T1, T2, T3, T4, or T5)-*egfp*, respectively.

### Transient Expression of Bm65 With Mutations in ^33^RRIK and ^76^KRKCSK Motifs

The mutation of 33R(A)34R(A)35I(A)36K(A) was introduced into Bm65 according to the instructions of the MutanBEST Kit (TaKaRa). Briefly, Bm65-F1 and Bm65-R were used to amplify Bm65 fragment for the generation of pMD18T-Bm65. Bm65M1-F and Bm65M-R were used to amplify the 33R(A)/Bm65 from pMD18T-Bm65 to produce pMD18T-33R(A)/Bm65. In a similar way, Bm65M2-F and Bm65M-R were used to amplify 33R(A)34R(A)/Bm65 from pMD18T-33R(A)/Bm65, Bm65M3-F and Bm65M-R were used to amplify 33R(A)34R(A)35I(A)/Bm65 from pMD18T-33R(A)34R(A)/Bm65. Bm65M4-F and Bm65M-R were used to amplify 33R(A)34R(A)35I(A)36K(A)/Bm65 from pMD18T-33R(A)34R(A)35I(A)/Bm65.

The mutation of 76K(A)77R(A)78K(A)CS81K(A) was introduced into Bm65 according to the instructions of the MutanBEST Kit (TaKaRa). Firstly, Bm65M5-F and Bm65-R4 were used to amplify 76K(A)/Bm65 from pMD18-T-Bm65. In a similar way, Bm65M6-F and Bm65-R4 were used to amplify 76K(A)77R(A)/Bm65 from pMD18T-76K(A)/Bm65. Bm65M7-F and Bm65-R5 were used to amplify 76K(A)77R(A)78K(A)/Bm65 from pMD18T-76K(A)77R(A)/Bm65, Bm65M8-F and Bm65-R6 were used to amplify 76K(A) 77R(A) 78K(A) 81K(A)/Bm65 from pMD18T-76K(A)77R(A)78K(A)/Bm65. Additionally, Bm65-F4 and Bm65-flag-R were used to amplify the cassette of P_Bm__65_-Bm65-flag from Bm-Bacmid, which was further mutated into P_Bm__65_-Bm65(M2)-flag at sites 76K(A), 77R(A), 78K(A), and 81K(A) of Bm65 in a similar way. To further study the effect of single point mutation in the ^76^KRKCSK motif on nuclear import of Bm65, 77R(A), 78K(A), and 81K(A) was introduced into Bm65 by PCR amplification from pMD18T-Bm65 with Bm65M9-F and Bm65-R7, Bm65M10-F and Bm65-R8, and Bm65M11-F and Bm65-R9, respectively.

Each mutant *Bm65* sequence was isolated from the corresponding recombinant plasmid by digestion, and the resulting DNA fragments were purified and subcloned into vector pFastHTB-P_ie__1_-*ns1*-*egfp* to generate the final plasmids. All primers used in the study are listed in Table 1.

**TABLE 1:** Primers, plasmids, and viruses used in the study.

**Primers**	**Primer sequence (5′–3′)**	**Enzyme digestion site**	**Plasmids**	**Viruses**
Bm65-F1	ATGAATTCATGGCGACG ACTCTGTACACCA	*Eco*RI	pFastHTB-P_Bm65_-Bm65-*egfp*	vBm^(PBm65–Bm65–egfp)^
Bm65-F2	ATGAATTCATGCAGCATTC GAACAAACAAG	*Eco*RI	pFastHTB-P_ie1_-*ns1*-*egfp*	vBm^Bm65KO–GFP^
Bm65-F3	ATGAATTCATGGAATACAA TCTTAAGCGTAAAT	*Eco*RI	pFastHTB-P_ie1_-Bm65-*egfp*	vBm^Bm65(M1)–Flag–GFP^
Bm65-F4	CGTACGTAAAACAGAGCGCGAATCA TGAGTTTAAAAGAGAA	*Sna*BI	pFastHTB-P_ie1_-Bm65(T1)-*egfp*	vBm^Bm65(M2)–Flag–GFP^
Bm65-R	ATCTGCAGCAACTTATTTGCTA ACAGAAATTTATGCA	*Pst*I	pFastHTB-P_ie1_-Bm65(T2)-*egfp*	vBm^Bm65–Flag–GFP^
Bm65-flag-R	TACTCGAGTTACTTATCGTCGTCATCCTT GTAATCCAACTTATTTGCTAACA	*Xho*I	pFastHTB-P_ie1_-Bm65(T3)-*egfp*	vBm^Bm65–GFP^
Bm65-R1	ATCTGCAGTTTGAAATACTTGC TGCATTTACGC	*Pst*I	pFastHTB-P_ie1_-Bm65(T4)-*egfp*	vBm^GFP–Bm65^
Bm65-R2	ATCTGCAGAAGATTGTATTCCATGCGGGC	*Pst*I	pFastHTB-P_ie1_-Bm65(T5)-*egfp*	
Bm65-R3	ATCTGCAGGTTAAGGTTGCTCGTGATGCC	*Pst*I	pMD18T-33R(A)/Bm65	
Bm65M1-F	ACCTTAACGCACGCATAAAACAGCA	—	pMD18T-33R(A)34R(A)/Bm65	
Bm65M2-F	ACCTTAACAGAGCCATAAAACAGCATTCGA	—	pMD18T-33R(A)34R(A)35I(A)/Bm65	
Bm65M3-F	ACCTTAACAGACGCGCAAAACAGCA	—	pMD18T-33R(A)34R(A)35I(A36K(A))/Bm65	
Bm65M4-F	ACCTTAACAGACGCATAGCACAGCATTC	—	pMD18T-76K(A)/Bm65	
Bm65M-R	TGCTCGTGATGCCCGTGTACAATTT		pMD18T-76K(A)77R(A)/Bm65	
Bm65M5-F	GGAATACAATCTTGCGCGTAAATGCAG		pMD18T-76K(A)77R (A)78K(A)/Bm65	
Bm65M6-F	GGAATACAATCTTGCGGCCAAATGC		pMD18T-K(76A)R(77A)K(78A)K(81A)/Bm65	
Bm65-R4	ATGCGGGCGGCGGTTTTGTAGT		pMD18T-Bm65	
Bm65M7-F	GCATGCAGCAAGTATTTCAAATTGC		pUC18-65US-Cm-65DS	
Bm65-R5	GGCCGCAAGATTGTATTCCAT		pFastHTB-Pie1-egfp-sv40-PH	
Bm65M8-F	GCGTATTTCAAATTGCGTCTCATCAAAGCCA		pFastHTB-Pie1-egfp-sv40-P_Bm65_-Bm65-flag	
Bm65-R6	GCTGCATGCGGCCGCAAGATTG		pFastHTB-P_Bm65_-Bm65-flag	
Bm65M9-F	GCCAAATGCAGCAAGTATTTCAAAT		HTB-Pie1-egfp-sv40-P_Bm65_-12C(A)46C(A)79C(A)/Bm65-flag	
Bm65-R7	CTTAAGATTGTATTCCATGCGGGC		pMD18T-P_Bm65_-Bm65-flag	
Bm65M10-F	GCATGCAGCAAGTATTTCAAATTGC		pMD18T-P_Bm65_-12C(A)/Bm65-flag	
Bm65-R8	ACGCTTAAGATTGTATTCCATGCG		pMD18T-P_Bm65_-12C(A)46C(A)/Bm65-flag	
Bm65M11-F	GCATATTTCAAATTGCGTCTCATCA		pMD18T-P_Bm65_-12C(A)46C(A)79C(A)/Bm65-flag	
Bm65-R9	GCTGCATTTACGCTTAAGATTGTATTC		pFastHTB-Pie1-*egfp*-sv40-P_Bm65_-Bm65(M2)-flag	
EGFP-F	ATGAATTCATGGTGAGCAAGGGCGA	*Eco*RI	pFastHTB-P_ie1_-*egfp*-*Bm65*	
EGFP-R	ATGGATCCCTTGTACAGCTCGTCCATG	*Bam*HI	pUC118-*egfp*	
Bm65-F5	ATGGATCCGCGACGACTCTGTACA	*Bam*HI	pUC118-*egfp-Bm65*	
Bm65-R10	GTAAGCTTTTACAACTTATTTGCT AACAGAAAT	*Hin*dIII	pFastHTB-P_ie1_-*egfp-Bm65*	
65US-F	ATAAGCTTCTCAAGCACGCCACTCTGC			
65US-R	TACTGCAGTTTTCCATTGTCCTGCCC			
Cm-F	GGATCCCTTCGAATAAATACCTGTGA			
Cm-R	CTGCAGAACCAGCAATAGACATAAGC			
65DS-F	TAGGATCCGCGAGCGCGTACGACT			
65DS-R	AAGGTACCCATGTACTTGCTCCACAGACTG			
C12-F	TGGGCCGTGTACATTCTGCGGCA			
C12-R	CACCTTGTTGGTGTACAGAGTCGTCGCCA			
C46-F	AAGGCTTTGCGCAACGCAACCA			
C46-R	GGCGCCTTGTTTGTTCGAATGCTGT			
C79-F	AAAGCCAGCAAGTATTTCAAATTGCGTCT			
C79-R	ACGCTTAAGATTGTATTCCATGCGG			

*Underlined letters indicate restriction enzyme digestion sites.*

### Preparation of Recombinant Viruses

Construction of *Bm65*-deleted Bm-bacmid (Bm^Bm65KO^) was performed as previously described (Tang et al., 2013). Colonies resistant to chloramphenicol, ampicillin, and kanamycin were selected for PCR confirmation with 65US-F/65US-R, Cm-F/Cm-R, and 65DS-F/65DS-R (data not shown). Deletion of *Bm65* and correct insertion of *Cm* cassette were named Bm^Bm65KO^. To generate a flag-tagged *Bm65* repair Bm-bacmid, the fragment containing *Bm65* and its native promoter sequence tagged with flag coding sequence at the 3′ end was amplified from Bm-bacmid by PCR with Bm65-F4 and Bm65-flag-R. The DNA fragment was subcloned into pFastHTB-Pie1-*egfp*-sv40-PH (Li et al., 2014) and pFastHTB (Invitrogen) digested with *Sna*BI and *Xho*I to generate pFastHTB-Pie1-*egfp*-sv40-P_Bm__65_-*Bm65*-flag and pFastHTB-P_Bm__65_-*Bm65*-flag, respectively. P_ie__1_-*egfp*-sv40-P_Bm__65_-Bm65-flag was transferred into the polyhedrin locus of the Bm^Bm65KO^, and the resulting Bm^Bm65–Flag–GFP^ was selected by blue–white screening and further confirmed by PCR with M13 primers.

Transposition between pFastHTB-Pie1-*egfp*-sv40-PH and Bm^WT^ or Bm^Bm65KO^ was made to generate Bm^WT–GFP^ and Bm^Bm65KO–GFP^, respectively. Transposition between HTB-P_ie__1_-egfp-sv40-P_Bm__65_-12C(A)46C(A)79C(A)/Bm65-flag and Bm^Bm65KO^ was made to produce Bm^Bm65(M1)–flag–GFP^, which contains mutations in the three cysteine sites of Bm65. The donor plasmid of HTB-P_ie__1_-*egfp*-sv40-P_Bm__65_-12C(A)46C(A)79C(A)/Bm65-flag was generated as follows. Briefly, P_Bm__65_-*Bm65*-flag was ligated into pMD18T to generate pMD18T-P_Bm__65_-*Bm65*-flag, which was used to amplify P_Bm__65_-12C(A)/Bm65-flag with C12-F and C12-R. C46-F and C46-R were used to amplify P_Bm__65_-12C(A)46C(A)/Bm65-flag from pMD18T-P_Bm__65_-12C(A)/Bm65-flag. Finally, P_Bm__65_-12C(A)46C(A)79C(A)/Bm65-flag was amplified with C79-F and C79-R from pMD18T-P_Bm__65_-12C(A)46C(A)/Bm65-flag. P_Bm__65_-12C(A)46C(A)79C(A)/Bm65-flag was ligated into pFastHTB-Pie1-*egfp*-sv40-PH digested with *Sna*BI and *Xho*I to generate HTB-Pie1-*egfp*-sv40-P_Bm__65_-12C(A)46C(A)79C(A)/Bm65-flag.

Recombinant viruses of vBm^Bm65(M2)–GFP^, vBm^Pie1–Bm65–EGFP^, and vBm^ Pie1–EGFP–Bm65^ were constructed as follows. Briefly, P_Bm__65_-Bm65(M2)-flag was used as template for amplification with Bm65-F2 and Bm65-flag-R. P_Bm__65_-Bm65(M2)-flag was ligated into pFastHTB-Pie1-egfp-sv40-PH digested with *Sna*BI and *Xho*I to generate pFastHTB-Pie1-egfp-sv40-P_Bm__65_-Bm65(M2)-flag. pFastHTB-P_ie__1_-*Bm65*-*egfp* was used to construct recombinant pFastHTB-P_ie__1_-*egfp*-*Bm65*. Briefly, EGFP-F and EGFP-R were used first to amplify the *egfp* fragment, which was subsequently ligated into pUC118 digested with *Eco*RI and *Bam*HI to generate pUC118-*egfp.* Subsequently, Bm65-F5 and Bm65-R10 were used to amplify the *Bm65* fragment, which was ligated with pUC118-*egfp* digested with *Bam*HI and *Hin*dIII to generate pUC118-*egfp-Bm65*. Finally, the purified *egfp-Bm65* from pUC118-*egfp-Bm65* was ligated into pFastHTB-P_ie__1_-*Bm65*-*egfp* digested with *Eco*RI and *Hin*dIII to generate the final pFastHTB-P_ie__1_-*egfp-Bm65*. Competent *E. coli* DH10B cells containing Bm^Bm65KO^ were transformed with pFastHTB-P_ie__1_-*Bm65*-*egfp*, pFastHTB-P_ie__1_-*egfp-Bm65* and pFastHTB-Pie1-*egfp*-sv40-P_Bm__65_-*Bm65*(M2)-flag, to produce Bm^Bm65(M2)–Flag–GFP^, Bm^Bm65–GFP^, and Bm^GFP–Bm65^, respectively.

### Transfection and Fluorescence Microscopy

BmN cells (10^6^ cells/well) were seeded into six-well culture plates and incubated at 27°C for 16–24 h before transfection. Recombinant DNA molecules (2 μg/well) and 5 μl Cellfectin (Invitrogen Life Technology) were mixed in 200 μl TC-100 serum free medium and incubated at 27°C for 45 min. Then, 800 μl serum free medium was added into the DNA-Cellfectin solution, which was finally overlaid onto BmN cells and incubated at 27°C for 5 h. After the incubated cells were washed with serum free TC-100 medium, 2 ml TC-100 medium containing 10% fetal bovine serum was added into each well for further culture. At 96 h post-transfection (hpt), the BV-enriched culture supernatants were harvested for further study. Fluorescence in BmN cells was observed through fluorescence microscopy (Olympus-IX73-DP80, Japan) at selected time points for further analysis. The fluorescent signal in each picture was counted one by one through observation by naked eye. The fluorescent cells were counted in each of 289 mm^2^ figure and 15 field of view were used for counting, which was used to make the statistical analysis using *t*-test.

### Western Blotting

Recombinant viruses (vBm^Bm65–Flag–GFP^, vBm ^Bm65(M1)–Flag–GFP^, vBm^Bm65(M2)–Flag–GFP^, vBm^Bm65–EGFP^, and vBm^EGFP–Bm65^) were used to infect BmN cells (10^6^/35-mm dish), which were harvested at 0, 3, 6, 12, 24, 48, 72, and 96 h post-infection (hpi). The cell pellets were respectively resuspended in 200 μl of RIPA Lysis Buffer (50 mM Tris [pH 7.4], 150 mM NaCl, 1% Triton X-100, 1% sodium deoxycholate, 0.1% SDS [Beyotime]), and protein concentrations of cell lysates were determined with BCA Protein Assay kit (Pierce) according to the manufacturer’s instructions. Total protein (30 μg) in each sample was dissolved in sample buffer (10 mM Tris-HCl pH 8.0, 1% SDS, 10% Glycerol, 0.008% Bromophenol Blue) with or without 5% (v/v) β-mercaptoethanol, which was subjected to SDS-PAGE and further transferred onto polyvinyldifluorene (PVDF) membranes (Millipore) as previously described (Yao et al., 2019).

The blots were blocked with 5% non-fat milk in 1× PBST (2.6 mM KCl, 0.136M NaCl, 8 mM Na_2_HPO_4_, 2 mM KH_2_PO_4_, 0.1% Tween 20, pH 7.4) overnight at 4°C and then incubated with antibodies against flag tag (Code#HT201, TransGen Biotech) at a dilution of 1:2000 in 5% non-fat milk in 1× PBST. After incubation with primary antibody, the blots were washed with 1× PBST for 10 min, which was repeated three times. Subsequently, the blots were incubated with goat anti-rat IgG conjugated to horseradish peroxidase (HS201-01, TransGen Biotech) diluted 1:5000 for 1 h at room temperature. The hybridization signal was visualized using enhanced chemiluminescence (ECL) (Amersham).

### Confocal Microscopy

Confocal microscopy was performed as previously described (Li et al., 2015; Tang et al., 2015), with some modifications. Briefly, BmN cells (1 × 10^5^) were seeded into a 35-mm glass-bottom cell culture dish (NSET). The cells were transfected with 2 μg recombinant plasmids, which can transiently express Bm65 and Bm65 truncations fused with EGFP under the control of *ie1* promoter. The cell culture supernatants were removed at 48 hpt and the cells were used for subcellular localization analysis of Bm65. Additionally, BmN cells (1 × 10^5^) infected with vBm^Bm65(M2)–GFP^ and vBm^Bm65–GFP^ were also used to study the subcellular localization of Bm65. The cell culture supernatants were removed at 3, 6, 12, 24, 48, and 72 hpi, respectively. Subsequently, these BmN cells were washed with PBS (2.6 mM KCl, 0.136M NaCl, 8 mM Na_2_HPO_4_, 2 mM KH_2_PO_4_, pH 7.4), fixed with 4% paraformaldehyde for 15 min, washed three times with PBS for 10 min, and permeabilized in 0.1% Triton X-100 for 15 min. Finally, the cells were stained with DAPI (60 μg/ml, Sigma) for 10 min, and washed three times with PBS. In addition, BmN cells (1 × 10^5^) infected with vBm^Bm65(M2)–Flag–GFP^ and vBm^Bm65–Flag–GFP^ were used for subcellular localization of Bm65 by immunocytochemistry assay. Briefly, BmN cells were treated with monoclonal antibodies (TransGen Biotech) against Flag, followed by treatment with secondary antibody conjugated-FITC (Alexa Fluor 647^®^, ab150107) and the nucleus was treated with DAPI (60 μg/ml, Sigma).

The cells were directly observed and photographed using Confocal Laser Scanning Microscopy (Model:TCS SP5 II, Leica corporation of Germany). Laser source is single-laser excitation source for multicolor and fluorescence emission was excited at 488 and 345 nm to detect the fluorescence signal of EGFP and DAPI, respectively. Glycerol objective (× 63) was used for imaging.

### Analysis of Viral Growth Curve

To further study whether deletion of Bm65 and alanine mutations in the ^76^KRKCSK motif affected production of recombinant virus, a virus growth curve analysis was performed as described previously (Tang et al., 2013). A monolayer of BmN cells (10^6^) was seeded in six-well plates for transfection with 2.0 μg of bacmid DNA and the experiments were repeated three times. The supernatants of transfected cells containing recombinant viruses were harvested at selected time points. The 50% tissue culture infective dose (TCID_50_) end-point dilution was carried out to determine the titer of BV as described previously, with modifications (O’Reilly et al., 1994). Viral infections were characterized by observation of fluorescence in BmN cells. It was identified to be positive if green fluorescence was observed in one or more BmN cells by fluorescence microscopy. Statistical analysis was performed using single factor analysis of variance.

## Results

### Comparison of the Amino Acid Sequences of Bm65 Homologs

**FIGURE 1:**
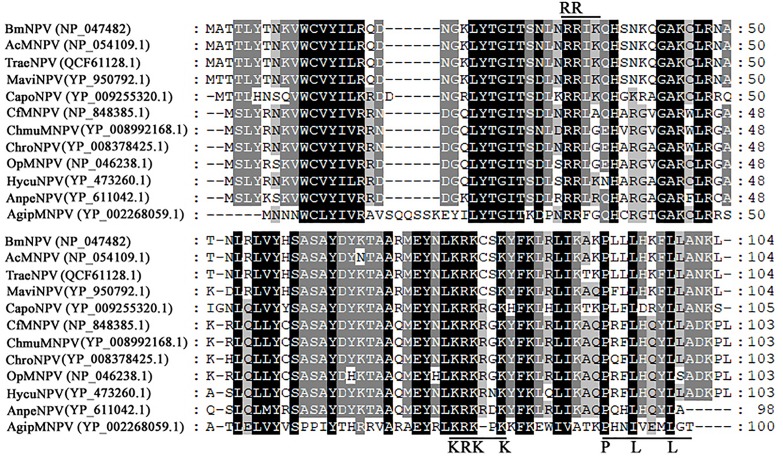
Alignment of Bm65 and its homologs. The alignment was performed using Clustal W and edited using Genedoc software. Identical amino acids are denoted by black shading and similar amino acids are denoted by gray shading. These sequences are from GenBank, and the accession numbers are as follows: BmNPV (NP_047482.1), AcMNPV (NP_054109.1), TraeNPV (QCF61128.1), MaviNPV (YP_950792.1), CfMNPV (YP_950792.1), ChmuMNPV (YP_008992168.1), ChroNPV (YP_008378425.1), OpMNPV (NP_046238.1), HycuNPV (YP_473260.1), AnpeNPV (YP_611042.1), CapoNPV (YP_009255320.1), and AgipMNPV (YP_002268059.1).

Bm65 codes for 104 deduced amino acid residues with a putative molecular mass of 12.2 kDa. Bm65 is not a core gene existing in all baculoviruses. Alignment result revealed that it is a highly conserved gene in baculoviruses of the genera Alphabaculovirus and Betabaculovirus (Figure 1). Ac79 [*orf*79 of Autographa californica multiple nucleopolyhedrovirus (AcMNPV)], a homologs of Bm65, has been reported to be an early gene involved in efficient budded virus production (Wu and Passarelli, 2012). Tang et al. (2015) reported that a conserved motif of GIY–YIG nuclease superfamily was found in the sequence of Bm65. Moreover, two possible typical nuclear localization signal (NLS) motifs of ^33^RRIK and ^76^KRKCSK are contained in the middle part of Bm65 sequence, and alignment result revealed that they are conserved in the Bm65 sequence and its homologs, Additionally, a leucine-rich motif of ^92^PLLLHKFLL in the C-terminal part of Bm65 sequence was found to be conserved in the multiple sequences, which was predicted to be a potential nuclear export signal by analysis of the online tool^1^.

### Analysis of Recombinant Bm65 Expressed in BmNPV-Infected BmN Cells

**FIGURE 2:**
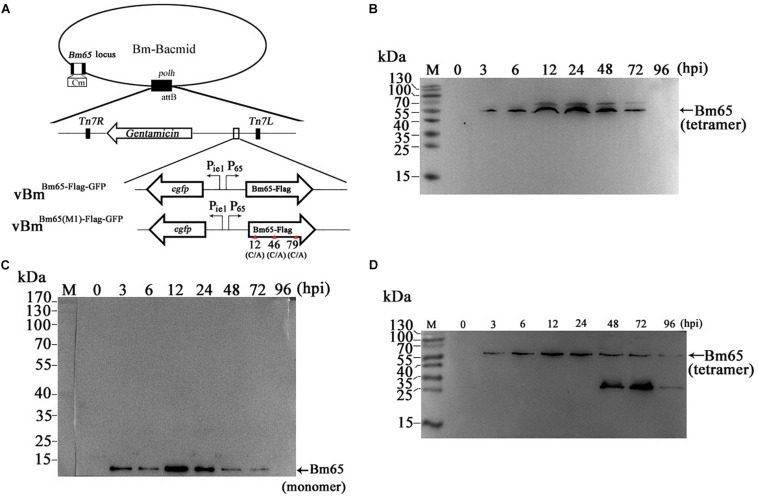
Western blotting analysis of Bm65-flag and Bm65(M1)-flag using antibodies against flag (Code#HT201 TransGen Biotech). **(A)** Strategy for expression of recombinant protein Bm65-flag and Bm65(M1)-flag. **(B)** Western blotting analysis of Bm65-flag expressed in BmNPV-infected BmN cells. **(C)** Western blotting analysis of Bm65-flag expressed in virus-infected BmN cells treated with β-mercaptoethanol. **(D)** Western blotting analysis of Bm65(M1)-flag expressed in virus-infected BmN cells. The prestained protein standards are on the left. The virus of vBm^Bm65(M1)–Flag–GFP^ used is one in which all three cysteines were changed to alanine.

Construction of recombinant virus for expression of Bm65 is described as Figure 2A. Flag-tagged Bm65 was examined from 3 to 72 hpi under the control of its natural promoter (Figures 2B–D). Western blotting analysis of total protein from BmN cells infected with vBm^Bm65–flag–GFP^ or vBm^Bm65(M1)–Flag–GFP^ was carried out. A specific protein band was examined from 3 to 72 hpi (Figure 2B). However, the experimentally determined mass of Bm65 was about four times as large as that of the predicted size of Bm65, indicating that Bm65 exists with a mainly tetrameric form in BmNPV-infected BmN cells. Protein lysate from BmN cells infected with vBm-bacmid^Bm65–Flag–GFP^ was treated with β-mercaptoethanol, which was subsequently subjected to Western blotting analysis. Only a Bm65 specific protein band migrating at only a size of approx 12 kDa was observed from 3 to 72 hpi (Figure 2C), showing that treatment of β-mercaptoethanol transformed the tetrameric form of Bm65 into a monomer. To explore the formative mechanism of Bm65 tetramer, three cysteine residues at positions 12, 46, and 79 of Bm65 were substituted by alanine residues and the mutant name fusion with flag was defined as Bm65(M1)-flag. The total protein from BmN cells infected with recombinant virus for expression of Bm65 mutant was determined by Western blotting. Only the tetrameric form of Bm65 was detected from 3 to 96 hpi (Figure 2D), indicating that the mutations did not prevent the formation of Bm65 tetramer. Additionally, extra-bands of about 26 kDa appear in Panel D from 48 to 72 hpi. They may be Bm65 dimer or Bm65 protein complex. Further experiments would be required to characterize this 26 kDa band. In conclusion, these results revealed that Bm65 is an early protein with a tetrameric form existing in BmNPV-infected BmN cells, but the formative mechanism requires further research.

### Subcellular Localization of Bm65 Truncations Fusion With EGFP

Bm65 is an early protein correlated with the repair of damaged DNA in nucleus of host cell (Tang et al., 2015, 2017). Therefore, it is indispensable for Bm65 to enter the nucleus of target cell for DNA repair. However, the mechanism of Bm65 translocation into nucleus remains unknown.

**FIGURE 3:**
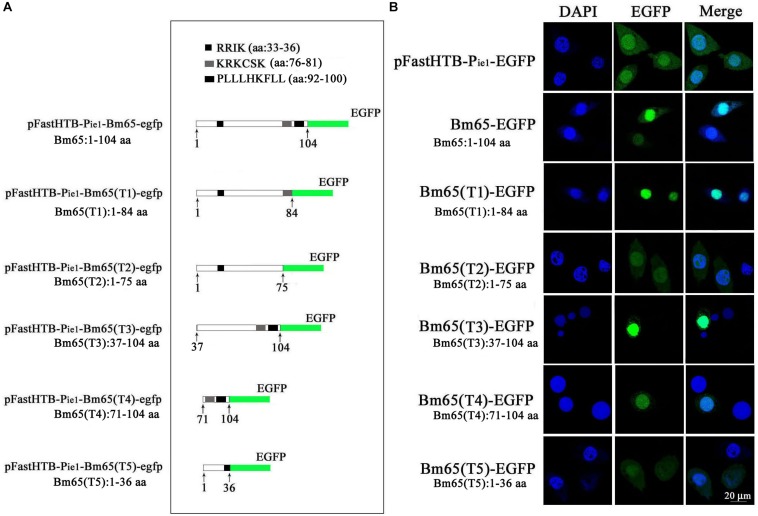
Confocal microscopy of truncated Bm65 fused with EGFP expressed in BmN cells. **(A)** Strategy for the construction of truncated Bm65 fusion with the N-terminal EGFP. Black boxes correspond to the basic residue cluster ^33^RRIK and a leucine-rich region ^92^PLLLHKFLL. Gray box and green boxes correspond to ^76^KRKCSK and EGFP, respectively. **(B)** Fluorescence microscopy of truncated regions of Bm65 fusion with EGFP expressed in BmN cells. The transient expression plasmids are indicated on the left. Fluorescence signal in BmN cells transfected with pFastHTB-P_ie1_-EGFP was used as a control.

Two possible NLS motifs of ^33^RRIK and ^76^KRKCSK, and a possible nuclear export signal of ^92^PLLLHKFLL are contained in the Bm65 sequence. To identify whether these putative motifs are functional, four truncations with the C-terminal and N-terminal deletions of Bm65 fusion with EGFP under control of *ie1* promoter were constructed to study intracellular distribution of Bm65, by observation of fluorescence signal. BmN cells were not infected in this assay. Transient expression assay showed that Bm65 was located mainly in the nuclei and only a little in the cytoplasm, but the intracellular distribution of truncated forms of Bm65 was correlated with the deletions of Bm65 (Figure 3). The truncated form of Bm65 (aa 1–84) accumulated almost exclusively in the nuclei of BmN cells. Compared with the truncated form of Bm65 (aa 1–84), a small part of green fluorescence was found in the cytoplasm except for the majority of green fluorescence accumulated in nucleus from the panel 2 of Figure 3 when BmN cells were transfected with the complete Bm65. The result indicated that the ^92^PLLLHKFLL motif may be involved with nuclear export of Bm65. The motif of ^92^PLLLHKFLL may be a functional nuclear export signal for transport of Bm65 from nucleus to cytoplasm. Further research is required to demonstrate the role of the ^92^PLLLHKFLL motif on nuclear export of Bm65. However, the truncated form (aa 1–75) of Bm65 was uniformly distributed within BmN cells when Bm65 with the C-terminal deletion of 29 aa, containing the motifs of ^92^PLLLHKFLL and ^76^KRKCSK, was expressed in BmN cells, indicating that ^76^KRKCSK may be a functional NLS for the active transport of Bm65 from cytoplasm to nucleus. Additionally, the truncated forms (aa 37–104 and aa 71–104) of Bm65 were accumulated mainly in the nucleus and only a little in the cytoplasm. The truncated form (aa 1–36) of Bm65 was evenly distributed in transfected BmN cells. The results indicated that the motif of ^33^RRIK has no effect on the nuclear import of Bm65, and the nuclear import of Bm65 is strictly dependent of the ^76^KRKCSK motif.

### Effect of Alanine Mutations in the Motifs of ^33^RRIK and ^76^KRKCSK on the Nuclear Import of Bm65

**FIGURE 4:**
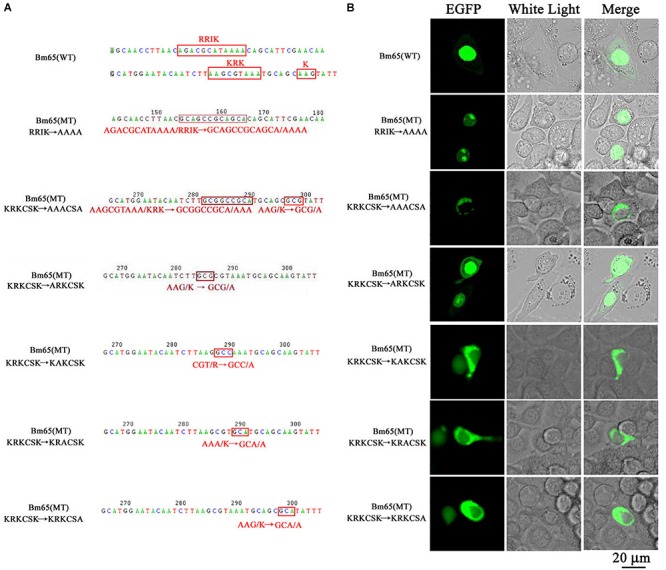
Effect of mutations on the subcellular localization of Bm65 expressed in BmN cells. **(A)** Strategy for construction of a series of transient expression vectors for expression of Bm65 mutant fusion with EGFP. **(B)** Intracellular distribution of green fluorescence in BmN cells transfected with a series of transient expression vectors. Plasmids used for transfection are indicated on the left. The numbers relative to the nucleotides position in DNA sequence were indicated above the sequence. The mutated codons in the sequence of *Bm65* were confirmed by sequencing and enclosed in red letters. Alanine mutations in the motifs of ^33^RRIK and ^76^KRKCSK are indicated below the sequence. MT, mutant type; WT, wild type.

The truncated form (aa 37–104) of Bm65 with deletion of the ^33^RRIK motif was found to be accumulated mainly in the nucleus, which is in line with the subcellular localization of Bm65 in BmN cells. The result indicated that the deletion did not affect the trafficking of Bm65 from cytoplasm to nucleus. To confirm this, alanine mutations were introduced into the ^33^RRIK motif of Bm65 to study the subcellular distribution of Bm65 through fluorescence observation. The ^33^AAAA (mutant 1) was accumulated mainly in the nucleus and some fluorescent aggregates were also present (Figure 4). However, the ^76^AAACSA (mutant 2) altered the pattern of subcellular localization of Bm65 in BmN cells, and the mutations severely blocked the transport of Bm65 from cytoplasm to nucleus. Fluorescence was accumulated exclusively in cytoplasm, and there were some fluorescent aggregates in the cytoplasm (Figure 4). According to the above results, it is believed that the motif of ^33^RRIK is not involved with the nuclear import of Bm65, but the motif of ^76^KRKCSK is an efficient NLS for transport of Bm65 from cytoplasm to nucleus.

To define the importance of basic amino acid residues in the motif of ^76^KRKCSK, a series of single point mutations, including ^76^ARKCSK (mutant 3), ^76^KAKCSK (mutant 4), ^76^KRACSK (mutant 5) and ^76^KRKCSA (mutant 6), were made to examine the subcellular localization of Bm65 by fluorescence microscopy. Mutant 3 did not block the nuclear import of Bm65, but other mutations impaired or blocked the nuclear import of Bm65 (Figure 3). Mutant 4 obviously impaired the nuclear import of Bm65, because some fluorescence was accumulated in the cytoplasm of some cells, and some fluorescence was found to be only in nucleus of some cells. The fluorescence of typically accumulated in cytoplasm for the mutation of mutant 4 was selected to describe the phenomenon of reduced nuclear entry (Figure 4). The result indicated that the residue ^77^R is involved with the efficiency of nuclear import. Mutant 5 and mutant 6 severely blocked the nuclear import of Bm65, and all fluorescence was accumulated dominantly in the cytoplasm.

In conclusion, these mutations resulted in the varied distribution pattern of green fluorescence in BmN cells compared with that of wild type Bm65. The intracellular distribution of fluorescence is summarized in Table 2. The results indicated that the ^76^KRKCSK motif is an efficient NLS for the final destination of Bm65, and the residues of ^78^K and ^81^K are essential for nuclear import of Bm65.

**TABLE 2:** Effect of ^33^RRIK and ^76^KRKCSK motifs on nuclear accumulation of Bm65.

**Mutants**	**Sequence and mutations**	**Subcellular localization**
WT	^33^RRIK, ^76^KRKCSK	N(++)/C
Truncation Truncation Truncation	Bm65 (aa 37–104) Bm65 (aa 1–84) Bm65 (aa 1–75)	N(++)/C N(++)/C N/C
Mutant 1	^33^AAAA	N(++)/C
Mutant 2	^76^AAACSA	C
Mutant 3	^76^ARKCSK	N(+)/C
Mutant 4	^76^KAKCSK	N(+)/C
Mutant 5	^76^KRACSK	C
Mutant 6	^76^KRKCSA	C

*N(++)/C, N(+)/C, N/C, and C indicated that fluorescence signal was predominantly but not mainly in the nucleus, in the nucleus was heavier than that of in the cytoplasm, equally between the nucleus and the cytoplasm, and mainly in the cytoplasm (C), respectively.*

### The ^76^AAACSA Mutant Blocked the Nuclear Import of Bm65

**FIGURE 5:**
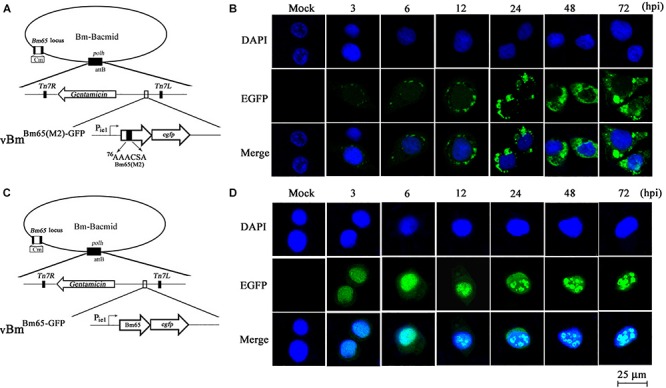
Subcellular localization of Bm65(M2)-GFP and Bm65-GFP fusion protein in virus-infected BmN cells. **(A)** Schematic diagram for construction of vBm^Bm65(M2)–GFP^. **(B)** Confocal fluorescence images of BmN cells infected with vBm^Bm65(M2)–GFP^. **(C)** Schematic diagram for construction of vBm^Bm65–GFP^. **(D)** Confocal fluorescence images of BmN cells infected with vBm^Bm65–GFP^. At 3, 6, 12, 24, 48 and 72 hpi, BmN cells were observed for fluorescence by confocal microscopy. From the top to the bottom: DAPI-treated nucleus, GFP-specific fluorescence and the merged images.

To further study the effect of the mutant 2 on the subcellular localization of Bm65 in virus-infected BmN cells, recombinant virus of vBm^Bm65(M2)–GFP^ (Figure 5A) was used to infect BmN cells for expression of mutant Bm65 EGFP fusion. Bm65 labeled EGFP was under the control of *ie1* promoter. The fluorescence signal was observed exclusively in the cytoplasm of virus-infected BmN cells at 3 hpi, and the fluorescence continued to be observed in cytoplasm from 6 to 72 hpi. Additionally, Bm65(M2)-GFP showed fluorescent aggregates in the form of punctate dots in the cytoplasm of virus-infected cells (Figure 5B). Together, these results demonstrated that alanine mutations including K76A, R77A, K78A, and K81A completely blocked the nuclear import of Bm65 in virus-infected BmN cells, and the motif of ^76^KKKCSK was confirmed to be an efficient NLS for transport of Bm65 from cytoplasm to nucleus.

vBm^Bm65–GFP^ was used as the control of wild type (Figure 5C). The result indicated that Bm65-GFP was accumulated mainly in the nucleus of virus-infected BmN cells, and some fulorescent aggregates were found to be accumulated in nucleus of virus-infected BmN cells (Figure 5D). Additionally, Tang et al. (2015) have examined the subcellular localization of wild type Bm65-EGFP by the observation of confocal microscopy. The results also confirmed that fluorescence signal and some fluorescenct aggregates were accumulated mainly in nucleus of virus-infected BmN cells. Compared with the localization of wild type Bm65, the mutant 2 was accumulated mainly in cytoplasm of virus-infected BmN cells. The results indicated that the motif of ^76^KKKCSK was an efficient NLS for the nuclear import of Bm65.

### Disruption of Intrinsic Stability of the Tetramer for Bm65 Fusion With EGFP

**FIGURE 6:**
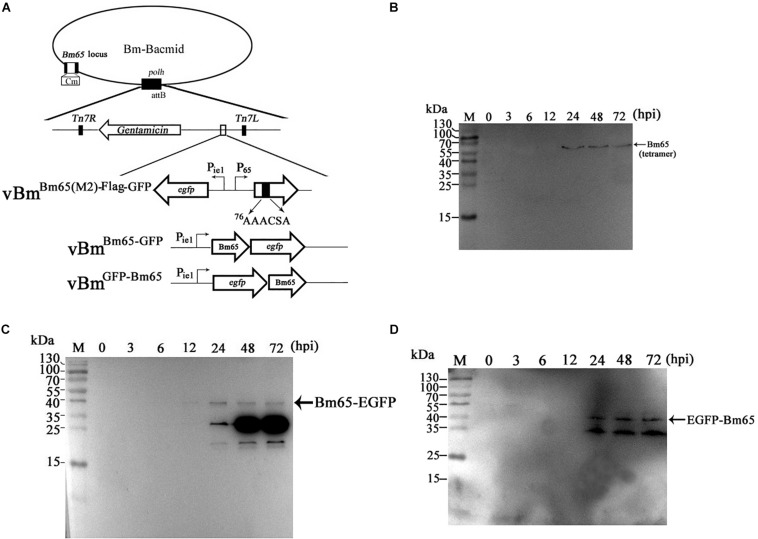
Effect of alanine mutations in the motif ^76^KRKCSK and Bm65 labeled with EGFP on Bm65 tetramer. **(A)** Strategy for construction of three recombinant viruses of vBm^Bm65(M2)–Flag–GFP^, vBm^Bm65–GFP^ and vBm^GFP–Bm65^. **(B)** Western blotting analysis of target protein from vBm^Bm65(M2)–Flag–GFP^ -infected BmN cells using the antibodies against the flag sequence. **(C)** Western blotting analysis of target protein from vBm^Bm65–GFP^-infected BmN cells using the antibodies against the EGFP sequence. **(D)** Western blotting analysis of target protein from vBm^GFP–Bm65^-infected BmN cells using the antibodies against the GFP sequence. The prestained protein standards are on the left. Times hpi are indicated above the lanes.

Due to some mutations in the ^76^KRKCSK NLS, Bm65 mutants were found to be accumulated exclusively in cytoplasm. To determine the effect of alanine mutations in the motif ^76^KRKCSK on the tetrameric form of Bm65, recombinant virus vBm^Bm65(M2)–Flag–GFP^ (Figure 6A) for expression of Bm65 mutant was made and used to infect BmN cells for Western blot analysis. Only a tetramer specific band of Bm65 was detected from 24 to 72 hpi (Figure 6B), and alanine mutations in the motif ^76^KRKCSK did not affect the formation of Bm65 tetramer. So, the tetrameric form of Bm65 can be produced in cytoplasmic and nuclear sites, but it was not required for the nuclear import of Bm65 because the monomer of Bm65-GFP also entered nucleus. According to the above results, it is regarded that the introduced mutations were not involved with the tetramer formation and/or on protein stability.

To further determine whether a fusion protein labeled with Bm65 produced the tetrameric form in BmN cells, recombinant viruses for expression of the N-terminal or C-terminal extension of Bm65 labeled with EGFP was under the control of *ie1* promoter, which were prepared and used to infect BmN cells for Western blotting analysis. A band migrating at 40 kDa, the size expected for a Bm65:EGFP fusion was detected from 24 to 72 hpi (Figures 6C,D) using antibodies against EGFP, and only the monomer form of Bm65 fusion, regardless of N-terminal or C-terminal, was formed. According to the result, it is regarded that EGFP fusion affect the stability the tetramer of Bm65.

### Immunocytochemistry Assay for Distribution of Bm65-Flag and Bm65(M2)-Flag

**FIGURE 7:**
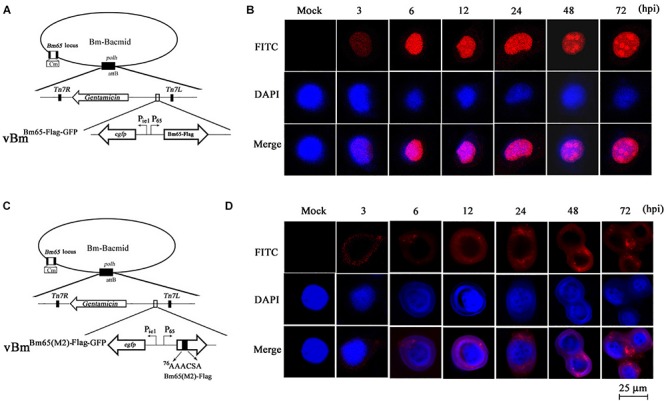
Subcellular localization of Bm65-flag and Bm65(M2)-flag in virus-infected BmN cells. **(A)** Schematic diagram for preparation of recombinant virus vBm^Bm65–Flag–GFP^. **(B)** Confocal fluorescence images of BmN cells infected with vBm^Bm65–Flag–GFP^. **(C)** Schematic diagram for preparation of recombinant virus vBm^Bm65(M2)–Flag–GFP^. **(D)** Confocal fluorescence images of BmN cells infected with vBm^Bm65(M2)–Flag–GFP^. At 3, 6, 12, 24, 48 and 72 hpi, cells were examined for red fluorescence by confocal microscopy. From the top to the bottom: red fluorescence, DAPI-treated nucleus and the merged images.

Although GFP-tagged Bm65 and its variants are sufficient to just prove nuclear localization ability of the NLS, Western blotting revealed that the recombinant virus expressing Bm65-GFP also expressed a large amount of extra proteins detected by anti-GFP antibody (Figures 6C,D). To preclude the possibility of fluorescent aggregates from the huge amount of truncated GFP and the mislocalization of Bm65 caused by GFP-tag, the immunocytochemistry assay was made in vBm^Bm65(M2)–Flag–GFP^ -infected BmN cells using antibodies against flag tag (Figure 7C). Meanwhile, vBm^Bm65–Flag–GFP^ was used as a control (Figure 7A). The immunocytochemistry assay of Bm65-flag was made in vBm^Bm65–Flag–GFP^-infected BmN cells using antibodies against flag, which was used as a control of wild type. The subcellular localization of Bm65 and its variant was judged from the distribution of red fluorescent signal observed by confocal microscopy.

The results indicated that Bm65-flag was accumulated mainly in the nucleus of BmNPV-infected BmN cells, and some fluorescent aggregates were found to be accumulated in nucleus of virus-infected BmN cells (Figure 7B). However, the mutant 2 was accumulated mainly in cytoplasm of virus-infected BmN cells and some fluorescent aggregates was found to be accumulated in cytoplasm (Figure 7D).

### Alanine Mutations in the Motif ^76^KRKCSK Sharply Impaired the Production of Infectious Virions

**FIGURE 8:**
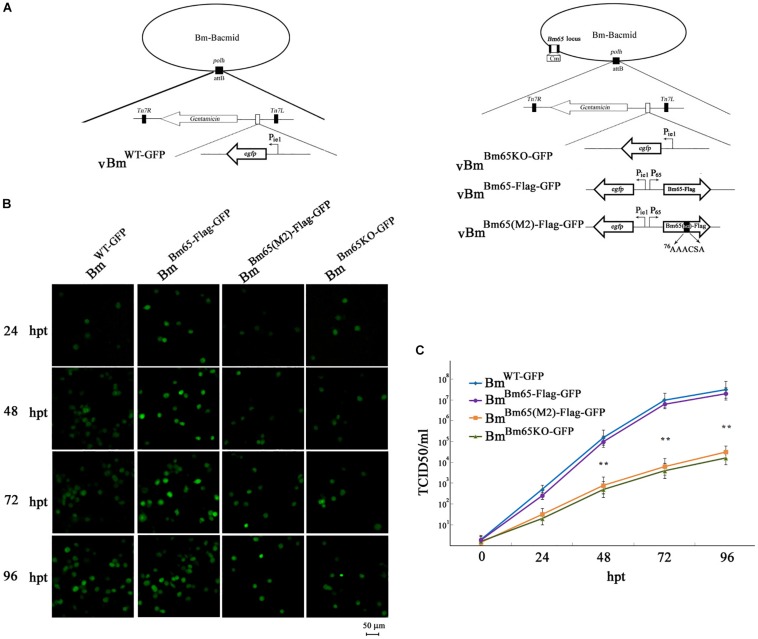
Analysis of viral replication in BmN cells. **(A)** Strategy for the construction of vBm^WT–GFP^, vBm^Bm65–Flag–GFP^, vBm^Bm65KO–GFP^ and vBm^Bm65(M2)–GFP^. **(B)** Fluorescence microscopy of BmN cells transfected with Bm^WT–GFP^, Bm^Bm65–Flag–GFP^, Bm^Bm65KO–GFP^, and Bm^Bm65(M2)–Flag–GFP^ at 24, 48, 72, and 96 hpt. The time after transfection was indicated on the left. **(C)** Virus growth curves generated from BmN cells transfected with Bm^WT–GFP^, Bm^Bm65–Flag–GFP^, Bm^Bm65KO–GFP^, and Bm^Bm65(M2)–GFP^. Transfection supernatants were harvested at selected time points and used for infectivity assays by the determination of TCID_50_. Each datum point represents the average value derived from three independent experiments (*n* = 3). Error bars indicate standard deviations. ^∗∗^ Indicates a statistically significant difference (*P* < 0.01).

To analyze whether *Bm65* is required for viral production, four recombinant Bm-Bacmids, including fully deleted-type (Bm^Bm65KO–GFP^), wild-type (Bm^WT–GFP^), repair-type 1 (Bm^Bm65–Flag–GFP^), and repair-type 2 (Bm^Bm65(M2)–Flag–GFP^) were generated and further transfected into BmN cells. Green fluorescence was observed by fluorescence microscopy in individual cultured cells as early as 24 hpt. However, in cells transfected with the Bm65KO and BM65(M2) constructs, the level of fluorescence was significantly lower than that of the control viruses (Figure 8B). Additionally, the transfected supernatants were harvested at 96 hpt, and used to infect BmN cells at a multiplicity of infection of 5. No difference was observed between the control viruses vBm^Bm65–Flag–GFP^ and vBm^WT–GFP^, but a 90% reduction on the fluorescence level was detected for vBm^Bm65KO–GFP^ and vBm^Bm65(M2)–Flag–GFP^, compared with the controls (data not shown). To confirm the role of Bm65 during viral propagation, the supernatants containing infectious virions were harvested from BmN cells transfected with each of the four bacmids at 24, 48, 72, and 96 hpt, and were used to infect BmN cells to compare the infectivity of vBm^WT–GFP^, Bm^Bm65KO–GFP^, vBm^Bm65–Flag–GFP^, and vBm^Bm65(M2)–Flag–GFP^ by analysis of the growth curves. The assay of TCID_50_ endpoint dilution on BmN cells was performed to determine BV titer. The results showed a steady increase in virus production and the same kinetics of growth for vBm^WT–GFP^ and vBm^Bm65–Flag–GFP^. In contrast, recombinant viruses of vBm^Bm65KO–GFP^ and vBm^Bm65(M2)–Flag–GFP^ showed reduced viral growth. According to the viral growth curve, the four viruses all showed an increased tendency in the slope of the growth curves, but the proliferation rate of vBm^Bm65KO–GFP^ and vBm^Bm65(M2)–Flag–GFP^ are obviously slower than that of the vBm^WT–GFP^ or vBm^Bm65–Flag–GFP^ (Figure 8C). Statistical analysis revealed that there was a significant difference of the viral titers between Bm65KO or BM65(M2) and Bm65 wild type virus from 48 to 96 hpi. Additionally, the viruses expressing GFP-tagged Bm65 and GFP-tagged Bm65 mutant 1 (^33^RRIK/AAAA) were constructed in our lab and they grew about as well as the wild type (Data not shown). The results confirmed that Bm65 is not essential for viral replication, but deletion of *Bm65* and alanine mutations in the motif ^76^KRKCSK significantly decreased the levels of infectious virions. As is well-known, the generation of progeny virions are involved with some steps, including virion assembly within nucleus, nuclear egress of assembled virions and budding from host cells. However, it is still unclear in which step was affected by the Bm65 mutant for the impairment of progeny production.

## Discussion

Nuclear proteins are translated from mRNA in cytoplasm and must be transported to nucleus where they exert their biological functions. It is generally accepted that a protein is targeted to nucleus by classical NLSs, which can be divided into two categories of monopartite and bipartite sequences. For example, the PKKKRKV sequence of simian virus 40 (SV40) large T antigen is a monopartite sequence (Kalderon et al., 1984), and the KRPAATKKAGQAKKKK sequence of nucleophosmin is a bipartite sequence (Robbins et al., 1991). The classical NLSs are directed to form a heterotrimeric complex including the cargo protein and two importins, which further mediates transport of the heterotrimer into nucleus by its affinity with nucleoporins (Ben-Efraim and Gerace, 2001). Some baculovirus proteins have also been reported to contain the classical NLSs. For example, the monopartite motif of classical NLSs was discovered in baculovirus polyhedrin and AcMNPV BV/ODV-c42 (Jarvis et al., 1991; Wang et al., 2008), and residues 804–827 of AcMNPV DNApol are a typical bipartite NLS motif (Feng and Krell, 2014). Additionally, some non-canonical NLSs (nNLSs) have also been identified in baculovirus proteins, which do not correspond with the prototypical NLS consensus sequence. For example, Guo et al. (2010) reported that two basic residue clusters at positions 117–120 (^117^RKRR) and 144–148 (^144^RKR-K) constituted an authentic NLS for mediating nuclear localization of Bm47. A novel localization signal containing K(75)/R(76), H(81), K(83)/R(84), R(87), and K(100) was scattered in the different positions of BmNPV LEF-11, which was identified to be critical for the nuclear localization of target protein (Zhang et al., 2014). The residues KKIREN of LEF-3 have been identified as a core NLS required for nuclear transport, which was further improved by other adjacent positively charged residues (Au et al., 2009).

Previous studies revealed that Bm65 accumulated mainly within the nucleus, and was involved with the repair of UVC-induced DNA damage (Tang et al., 2015, 2017). Moreover, Ac79, a homolog of Bm65, is responsible to encode an early gene product that are structurally similar to UvrC and intron-encoded endonucleases, which was required for efficient BV production (Wu and Passarelli, 2012). Alignment result showed 99% sequence identity with the amino acid of Bm65. The high similarity of sequence implied that the role of Bm65 in viral propagation may be similar to Ac79. However, the mechanism by which Bm65 is transported into the nucleus of virus-infected cells and the failure of nuclear import of Bm65 on viral production are still unclear. In the study, flag-labeled Bm65 is under the control of Bm65 native promoter in the study (Figures 2, 8), and it was detected from 3 to 72 hpi (Figures 2B–D). So, we think that Bm65 native promoter is similar to *ie1* promoter, and it can function like the *ie1* promoter. To accelerate the progress on Bm65 research, some plasmids containing *ie1* promoter such as pFastHTB-P_ie__1_-*ns1*-*egfp* have been constructed and conserved in our laboratory, which can be used for the transient expression analysis of Bm65 in BmN cells. So, *ie1* promoter was chosen to drive the expression based on the convenience and economic benefits in the study. A series of truncations and arginine/lysine-to-alanine mutations severely impaired the nuclear import of Bm65. The ^76^KRKCSK motif of Bm65 was identified as a novel NLS required for nuclear import of Bm65, but another basic cluster of ^33^RRIK was identified as unnecessary for the nuclear import of Bm65. Furthermore, the leucine-rich cluster of ^92^PLLLHKFLL may function as a nuclear export signal for maintaining the dynamic balance of Bm65 between nucleus and cytoplasm.

Previous research has revealed that *Bm65* is not essential for viral propagation (Ono et al., 2012). However, Tang et al. (2013) reported that Bm65 was an essential gene for viral propagation. In the present study, Bm65 was identified as an unnecessary gene for viral propagation. In Tang et al. (2013), Bm65 was regarded to be an essential gene for viral propagation, which may be resulted from the failure of transfection of BmN cells. As we know, donor plasmid of pFB-ieGP-Bm65 was transformed into electrocompetent DH10B cells containing Bm^Bm65KO^, which was used for the generation of Bm^Bm65KO–GP^ by transposition. Furthermore, the recombinant Bm^Bm65KO–GP^ extracted from DH10B cells was used for transfection of BmN cells. However, the pFB-ieGP-Bm65 was also extracted from DH10B cells besides the Bm^Bm65KO–GP^, and the abundance of pFB-ieGP-Bm65 is more than that of Bm^Bm65KO–GP^ in the extracted DNA. Fluorescence signal can be observed in BmN cells transfected with pFB-ieGP-Bm65, but no infectious virions were produced in the transfection supernatant. When the transfection supernatant was collected and further used for infection of BmN cells, it can not initiate the second infection of BmN cells and no fluorescence signal was observed in BmN cells. So, Tang et al. (2013) regarded that Bm65 was an essential gene for viral propagation by mistake. In fact, infectious virions can be produced in BmN cells transfected with Bm^Bm65KO–GP^. Furthermore, the recombinant virus with deletion of *Bm65* was confirmed to be infectious by passage analysis in BmN cells, but the analysis of viral titer showed that the deletion significantly decreased the production of infectious virions (Figure 8C). So, Bm65 was not an essential gene for viral propagation in the study, but it can regulate the production of viral propagation.

Furthermore, mutations in the motif of ^76^KRKCSK resulted in the cytoplasmic accumulation of Bm65. Compared with wild-type Bm65, the mutations greatly decreased the production of infectious virions. Bm65 was first identified to form a stable sodium dodecyl sulfate (SDS)-resistant tetramer in the natural state, and there was some fluorescent aggregates in cytoplasm and nucleus when alanine mutations were made in the motif of ^76^KRKCSK. Compared with wild type of Bm65 (Figure 5D), fluorescent aggregates caused by the mutant type of Bm65 are accumulated exclusively in the cytoplasm of virus-infected BmN cells (Figure 5B), and the fluorescent aggregates observed in wild type and mutant Bm65 virus-infected cells vary in the sizes and levels. It is supposed that Bm65 tetramer may increase the possibility of the formation of fluorescent aggregates. Of course, the misfolding of Bm65 is an important factor for the formation of protein aggregates. To the best of our knowledge, some baculovirus proteins such as LEF-10 and LEF-11 form protein aggregates (Dong et al., 2015; Xu et al., 2016). Moreover, amyloid β-protein (Walsh et al., 1999; Rangachari et al., 2007), sup35 (Kryndushkin et al., 2003), and α-synuclein (Rekas et al., 2010) are able to form SDS-resistant aggregates. Protein aggregation is a natural phenomenon that occurs both *in vitro* and *in vivo*. However, it is generally regarded to be harmful and has been implicated in a wide variety of diseases such as neurodegenerative diseases, and the possible correlations between protein aggregates and diseases deserves our deep consideration. Besides the tetrameric form from 3 to 96 hpi, a major lower molecular weight band at about 26 kDa was also examined in Figure 2D from 48 to 72 hpi, indicating that it may be a Bm65 dimer. The mutations of cysteine residues at positions 12, 46, and 79 did not affect the formation of Bm65 tetramer, but they directly resulted in increased instability of Bm65 tetramer in late infection. Additionally, some unexpected bands around 26 kDa and below 26 kDa were examined in Figures 6C,D. The abundance of the lower molecular weight proteins is so rich, but the synthesis mechanism is still unclear. Therefore, the possibility of Bm65 tetramer by disulfide linkage formation was precluded by the mutational analysis.

Additionally, recombinant plasmid of pET-30a-Bm65 was constructed by Tang et al. (2015) and transformed into BL21 cells for expression of 6 × His-Bm65. Western blotting result indicated that only 6 × His-Bm65 monomer was present in the soluble supernatant from the lysates of BL21 cells. Moreover, a lot of Bm65 inclusion body was found to be produced in *E. coli*. Therefore, we think that it is unfavorable for the formation of Bm65 tetramer in *E. coli* strains. Compared with the monomer in *E. coli*, Bm65 tetramer was examined in vBm^Bm65–Flag–GFP^-infected BmN cells, but GFP-tagged Bm65 monomer was examined in virus-infected BmN cells in the study (Figures 6C,D). It was regarded that flag tagged Bm65 is equivalent to the Bm65 native state, but the GFP tag affected the multimerization of Bm65. Moreover, the intermediate band about 26 kDa in Figure 2D from 48 to 72 hpi may be Bm65 dimer or Bm65 protein complex. The replacement of cysteine with alanine may decreased the stability of Bm65 tetramer, which was involved with the generation of the intermediate. Additionally, the formation of Bm65 tetramer may need the assistance from other proteins in BmNPV-infected BmN cells. Tang et al. (2015) reported the monomers-only, which was obtained in the soluble supernatant from the lysates of BL21 cells. In the study, Bm65 tetramer was easily produced in BmNPV-infected BmN cells, which has nothing to do with disulfide linkage. Some mutations in the motif of ^76^KRKCSK resulted in failure of the nuclear import of Bm65, which was involved with the decreased production of infectious virions. Moreover, we found that there are a lot of hydrophobic amino acids scattered in the sequence of Bm65, which may be the direct reason for the formation of Bm65 tetramer. Further research is required to disclose the formative mechanism of Bm65 tetramer. Meanwhile, it is also an interesting scientific issue for further investigation whether these hydrophobic amino acids are positively selected during virus/host coevolution.

## Data Availability Statement

The datasets generated for this study are available on request to the corresponding author.

## Author Contributions

GL designed the study, researched, and analyzed the data. XQ, HC, and LD researched the data. FC, ZG, and ZH contributed to the discussion. KC provided financial assistance. QT designed the study and wrote the manuscript.

## Conflict of Interest

The authors declare that the research was conducted in the absence of any commercial or financial relationships that could be construed as a potential conflict of interest.

## Footnotes

1 http://www.cbs.dtu.dk/services/NetNES/
